# Pre-existing *Schistosoma japonicum* infection alters the immune response to *Plasmodium berghei* infection in C57BL/6 mice

**DOI:** 10.1186/1475-2875-12-322

**Published:** 2013-09-14

**Authors:** Mei-lian Wang, Ya-ming Cao, En-jie Luo, Ying Zhang, Ya-jun Guo

**Affiliations:** 1 Department of Microbiology and Parasitology, College of Basic Medical Sciences, China Medical University, No. 92 Beier Road, Heping District, Shenyang 110001, China; 2 Department of Immunology, College of Basic Medical Sciences, China Medical University, No. 92 Beier Road, Heping District, Shenyang 110001, China; 3 Department of Sonography, Shengjing Hospital of China Medical University, No. 36 Sanhao Street, Heping District, Shenyang 110004, China

**Keywords:** *Plasmodium berghei* ANKA, *Schistosoma japonicum*, Co-infection, Inflammatory cytokine

## Abstract

**Background:**

Since helminths and malaria parasites are often co-endemic, it is important to clarify the immunoregulatory mechanism that occurs during the process of co-infection. A previous study confirmed that dendritic cells (DCs) are involved in the establishment and regulation of the T-cell-mediated immune response to malaria infection. In the current study, distinct response profiles for splenic DCs and regulatory T cell (Treg) responses were assessed to evaluate the effects of a pre-existing *Schistosoma japonicum* infection on malaria infection.

**Methods:**

Malaria parasitaemia, survival rate, brain histopathology and clinical experimental cerebral malaria (ECM) were assessed in both *Plasmodium berghei* ANKA-mono-infected and *S. japonicum-P. berghei* ANKA-co-infected mice. Cell surface/intracellular staining and flow cytometry were used to analyse the level of splenic DC subpopulations, toll-like receptors (TLRs), DC surface molecules, Tregs (CD4^+^CD25^+^Foxp3^+^), IFN-γ/IL-10-secreting Tregs, and IFN-γ^+^/IL-10^+^-Foxp3^-^CD4^+^ T cells. IFN-γ, IL-4, IL-5, IL-10 and IL-13 levels were determined in splenocyte supernatants using enzyme-linked immunosorbent assay (ELISA).

**Results:**

The co-infected mice had significantly higher malaria parasitaemia, compared with the mono-infected mice, on days 2, 3, 7 and 8 after *P. berghei* ANKA infection. *M*ono-infected mice had a slightly lower survival rate, while clinical ECM symptoms, and brain pathology, were significantly more severe during the period of susceptibility to ECM. On days 5 and 8 post *P. berghei* ANKA infection, co-infected mice had significantly lower levels of CD11c^+^CD11b^+^, CD11c^+^CD45R/B220^+^, CD11c^+^TLR4^+^, CD11c^+^TLR9^+^, CD11c^+^MHCII^+^, CD11c^+^CD86^+^, IFN-γ-secreting Tregs, and IFN-γ^+^Foxp3^-^CD4^+^ T cells in single-cell suspensions of splenocytes when compared with *P. berghei* ANKA-mono-infected mice. Co-infected mice also had significantly lower levels of IFN-γ and higher levels of IL-4, IL-5, and IL-13 in splenocyte supernatants compared to mono-infected mice. There were no differences in the levels of IL-10-secreting Tregs or IL-10^+^Foxp3^-^CD4^+^ T cells between co-infected and mono-infected mice.

**Conclusions:**

A Tregs-associated Th2 response plays an important role in protecting against ECM pathology. Pre-existing *S. japonicum* infection suppressed TLR ligand-induced DC maturation and had an anti-inflammatory effect during malaria infection not only by virtue of its ability to induce Th2 responses, but also by directly suppressing the ability of DC to produce pro-inflammatory mediators.

## Background

Malaria is an infectious disease caused by the *Plasmodium* parasite that continues to be a health issue for humans. It is one of the most common pathogenic factors of morbidity and mortality in sub-Saharan Africa [1]. More than one million children are dying each year as a result of malaria infection [2]. Experimental cerebral malaria (ECM), caused by infection with *Plasmodium berghei*, can result in cerebral inflammation and is a form of malaria that is life threatening in humans. The prominent pathogenesis of cerebral malaria (CM) is adherence and sequestration of parasitized red blood cells (pRBCs), immune cells and platelets to the vascular endothelial cells lining the small blood vessels of the brain. This leads to micro-haemorrhages and oedema in the brain [3,4].

Immunity to malaria has been studied extensively, however it is still not fully understood. It is generally thought that the balance between pro- and anti-inflammatory cytokines plays a very important role in the regulation of the immune response and pathogenesis [5]. A strong Th1 immune response to intracellular *Plasmodium* could prevent multiplication by this organism during the early stages of malaria infection, thus impacting the course of the disease. However, the body may then be exposed to severe immunopathology due to excessive production of pro-inflammatory cytokines (e g, interferon-γ, IFN-γ), combined with inadequate production of others (e g, interleukin-10, IL-10), constituting a passive effect. It is possible though, that the course of *Plasmodium* infection could be changed, if the balance of pro- and anti-inflammatory cytokines were broken, such as with a concomitant helminth infection.

Schistosome infections are common in many tropical regions of the world, ranking second only to malaria [4,6]. Three main *Schistosoma* species, *Schistosoma mansoni, Schistosoma japonicum,* and *Schistosoma haematobium*[7], frequently infect humans and these infections significantly impact public health. Recently, there has been an increasing awareness that helminth infections can ameliorate pro-inflammatory conditions due to their inherent ability to induce Th2 responses to various cytokines and pathways [8,9]. Kane *et al.* demonstrated that helminths had direct anti-inflammatory effects on innate immune responses. In that study, it was reported that the eggs of *S. mansoni* could suppress LPS-induced activation of immature murine dendritic cells (DCs) [10].

As helminths and malaria parasites are often co-endemic, schistosomiasis and malaria are frequently observed concomitantly. The existence of shared antigens and cross-reactive antibodies to different components of the two parasites has been confirmed in a previous report [6]. In the past few years more and more studies have been conducted to elucidate the immune mechanism(s) involved in worm and malaria co-infections. However, many of these studies have produced conflicting results, which has made it difficult to clearly understand the outcomes of these co-infections [11]. Some studies have reported an increased incidence of falciparum malaria in hosts with *S. mansoni*[12] while other studies have indicated that *S. haematobium* provides some protection from malaria (e g, lower parasitaemia, and lower incidence) [13,14]. These contradictory results may be caused by differences between different helminths and the infection stage of the parasites [5,15-17]. Currently, *S. mansoni* is the most widely used schistosome species for evaluating host immune responses [18]. To date, no reports on co-infections with *S. japonicum and Plasmodium* have been found. According to a previous report, *S. japonicum* infection was associated with more severe hepatic disease in humans than compared with *S. mansoni* infection [19]. It was suggested that there was a significant immunological difference between *S. japonicum* and *S. manson*i [19].

In the present study, *S. japonicum*, along with the *P. berghei* ANKA strain were used to produce a co-infection model in C57BL/6 mice. This model is likely a better fit for investigating the immunomodulatory mechanism of this co-infection in Southern Chinese populations since *S. japonicum* is the only schistosome species present in South China.

It has previously been demonstrated that Tregs can suppress Th1 responses to malaria infection by modifying DCs [20]. In the current study, pre-existing *S. japonicum* infection strengthened the Tregs-associated Th2 response to the malaria infection and this Th2 response played an important role in protecting against ECM pathology. In addition, *S. japonicum* infection suppressed the proliferation of DC subpopulations and weakened DC maturation and cytokine secretion. This indicated that pre-existing *S. japonicum* infection had anti-inflammatory effects during the malaria infection, not only by virtue of its ability to induce Th2 responses, but also by directly suppressing the ability of DCs to produce pro-inflammatory mediators.

## Methods

### Mice, parasites, and experimental infection

Female C57BL/6 mice, four weeks old, were purchased from the Beijing Animal Institute (Beijing, China). They were kept in the animal facility at China Medical University. Mice were maintained in individually ventilated cages and supplied with heat-sterilized food and distilled water *ad libitum*. The mice were randomly assigned to three groups. 25 mice were infected with *P. berghei* ANKA (*P. berghei* ANKA*-*mono-infection group), 15 mice were infected with *S. japonicum*. (*S. japonicum*-mono-infection group), and 25 mice were infected with both *S. japonicum* and *P. berghei* ANKA (co-infection group).

The *S. japonicum* strain was obtained from the Jiangsu Institute of Parasitic Diseases (Wuxi, China). A total of 50 cercariae of *S. japonicum* were administered percutaneously to C57BL/6 mice when the mice were five weeks of age.

*Plasmodium berghei* ANKA strain was provided by Dr Motomi Torii (Department of Molecular Parasitology, Ehime University Graduate School of Medicine, Ehime, Japan). Parasites were stored as frozen stabilates at −80°C. To obtain experimental inocula of *P. berghei* ANKA, pRBCs were sequentially passaged through three homologous donor mice. Infections were initiated in C57BL/6 mice by intraperitoneal (ip) injection of 1×10^6^*P. berghei* ANKA-pRBCs eight weeks after infection with *S. japonicum*.

Three mice each from the *P. berghei* ANKA-mono-infection group, the *S. japonicum-*mono-infection group, and the co-infection group were euthanized at 0, 3, 5, and 8 day post-*P. berghei* ANKA infection.

The current study has been reviewed and approved by China Medical University Institute of Medical Research Animal Ethics Committee.

### Confirmation of helminth infection

Helminth infection was confirmed by the presence of worms and liver granulomas upon necropsy. Worms were obtained by portal perfusion [21], and livers were examined for the presence of granulomas under a stereomicroscope.

### Malaria parasitaemia, survival rates and disease assessment

Parasitaemia was determined by light microscopy of Giemsa-stained, thin (tail) blood smears. Slides were coded and pRBCs were counted microscopically in at least five microscopic fields, each containing approximately 300 cells.

Mice were monitored for mortality daily, post*-P. berghei* ANKA infection, to evaluate the survival rate of *P. berghei* ANKA-mono-infected and the co-infected mice*.* Clinical ECM was also assessed and was defined by the presence of the following signs [22]: ruffled fur, hunching, wobbly gait, limb paralysis, convulsions, and coma. Each sign was given a score of 1. Animals with scores ≥4 were considered to have severe ECM.

### Spleen cell culture

Spleen cell culture was prepared as previously described [22,23]. Briefly, the spleen was aseptically removed from each mouse and pressed through a sterile fine-wire mesh with 10 ml of RPMI-1640 (Life Technologies, Shanghai, China) supplemented with 5% heat-inactivated fetal calf serum (FCS; Hyclone Laboratories, Inc, South Logan, Utah, USA), 25 mM Hepes (Life Technologies, Shanghai, China), 0.12% gentamicin (Schering-Plough, Kenny Worth, New Jersey, USA), and 2 mM glutamine (Life Technologies, Shanghai, China). Cell suspensions were centrifuged at 350 g for 10 min at room temperature (RT). Erythrocytes were lysed with cold 0.17 M NH_4_Cl and the cells were washed twice with fresh medium. The viability of the spleen cells was determined by trypan blue exclusion and was always >90%. Spleen cells were adjusted to a final concentration of 10^7^ cells/ml in RPMI-1640 supplemented with 10% heat-inactivated FCS. Aliquots (500 μl/well) of the cell suspension were incubated in 24-well flat bottom culture plates (Falcon®, Corning Life Sciences, CA, USA) in triplicate for 48 hr at 37°C in a humidified 5% CO_2_ incubator. The 24-well plates were then centrifuged at 350 g for 10 min at RT and the supernatants were collected and stored at −80°C until assayed for cytokine levels.

### Cytokine analysis

IFN-γ, IL-4, IL-5, IL-10 and IL-13 levels in splenocyte supernatants were measured using commercial enzyme-linked immunosorbent assay (ELISA) kits according to the manufacturer's protocol (R&D Systems, Minneapolis, MN, USA). The OD values were read in a microplate reader at 450 nm. The concentrations of cytokines in samples were calculated against the standard curve generated using recombinant cytokines.

### Cell surface/intracytoplasmic staining and flow cytometry

All flow cytometry analyses were performed on a FACS Calibur (BD Biosciences, San Diego, CA, USA) and analysed with Cell Quest software (version 3.3; BD Biosciences, CA, USA). All antibodies for FACS were purchased from BD Biosciences or e Bioscience, unless otherwise indicated.

For analysis of spleen DCs, single-cell suspensions of splenocytes were first pre-incubated with anti-mouse CD16/32 (2.4G2) monoclone antibody (mAb) to block Fc receptors and then stained with a combination of FITC-conjugated anti-mouse CD11c (clone HL3) mAb, PE-conjugated anti-mouse CD11b (clone M1/70) mAb, PerCP-conjugated anti-mouse CD45R/B220 (clone RA3-6B2) mAb, PE-conjugated anti-mouse MHC class II (clone M5/115.15.2) mAb, and PE-conjugated anti-mouse CD86 (clone GL1) mAb. Isotype-matched Abs were used as staining controls.

DC cell surface TLRs were also evaluated. For TLR4 analysis, splenocytes were harvested, blocked with anti-CD16 ⁄ CD32, and then stained using a combination of FITC-conjugated anti-mouse CD11c (clone HL3) and PE-conjugated anti-mouse TLR4 (MTS510).

For intracellular TLR9 staining of DCs, splenocytes were blocked with anti-CD16⁄CD32 after harvesting and then stained using FITC-conjugated anti-mouse CD11c (clone HL3). After fixation and permeabilization using staining buffer reagents as instructed by the manufacturer, cells were incubated with biotinylated anti-mouse TLR9 (clone 5G5, Hycult Biotechnology (HBT), Uden, The Netherlands) followed by PE-conjugated streptavidin (Biolegend, San Diego, CA, USA).

Spleen cells that were previously collected from C57BL/6 mice at different time points after infection and stimulated with ConA (5-10 ug/ml) for 48 hr and trypsinized during the final 8 hr were used to analyse Tregs and IL-10-secreting Tregs in the spleen CD4^+^ T cell population and to analyse the IL-10 levels produced by Foxp3^-^CD4^+^T cells. Cell concentration was then adjusted to 2×10^6^/ml, followed by a 4 hr stimulation with plate-bound anti-mouse CD3 (1 μg/ml) and anti-mouse CD28 (0.2 μg/ml), combined with Golgi Stop (Cat no 554724). After continued co-culture at 37°C for 4 hr, cells were washed with 3% FCS and re-suspended in 100 μl of 3% FCS. FITC-conjugated anti-mouse CD4 and PE- conjugated anti-mouse CD25 (clone 3C7) were added for surface staining. The cells were then fixed, permeabilized, and intracytoplasmic staining was performed using APC-conjugated anti-Foxp3 (clone FJK16s) and PerCP-Cy5.5-conjugated anti-IL-10 (clone JES5-16E3). FITC-conjugated rat IgG2b was used as the isotype control.

To analyse IFN-γ-secreting Tregs in the spleen CD4^+^ T cell population and to analyse IFN-γ produced by Foxp3^-^CD4^+^T cells, spleen cells, that had been previously collected from C57BL/6 mice at different time points after infection and stimulated with PMA and ionomycin for 2 hr at 37°C and then Golgi Stop (Cat no 554724), were added to each reaction (1:500 [v/v]). The next steps (co-culture, washing with FCS, re-suspending, surface staining with FITC-conjugated anti-CD4 and PE- conjugated anti-CD25) were carried out as described above. The cells were then fixed, permeabilized, and intracytoplasmic staining was performed using APC-conjugated anti- Foxp3 (clone FJK16s) and PerCP-Cy5.5-conjugated anti-IFN-γ. FITC-conjugated rat IgG2b was used as the isotype control.

### Histopathology

Immediately after death, the brains of the mice were removed and fixed in 1% buffered formalin for 48 hr. The brain tissue was then dehydrated using graded alcohols and xylene, and then embedded in paraffin. Continuous coronal sections of the tissue were made using a microtome. Five randomly selected sections were made into slides and stained with hemalaun eosin. The haemorrhage and the infiltration of immune cells were then examined in sections from the co-infected, *P. berghei* ANKA -mono-infected, and the normal control mice.

### Statistical analysis

Data were analysed using Prism (GraphPad, La Jolla, CA). Independent-samples *t*-tests were performed. All tests were considered significant when *P* <0.05.

## Results

### Parasitaemia, survival rate and disease assessment of ECM

**Figure 1:**
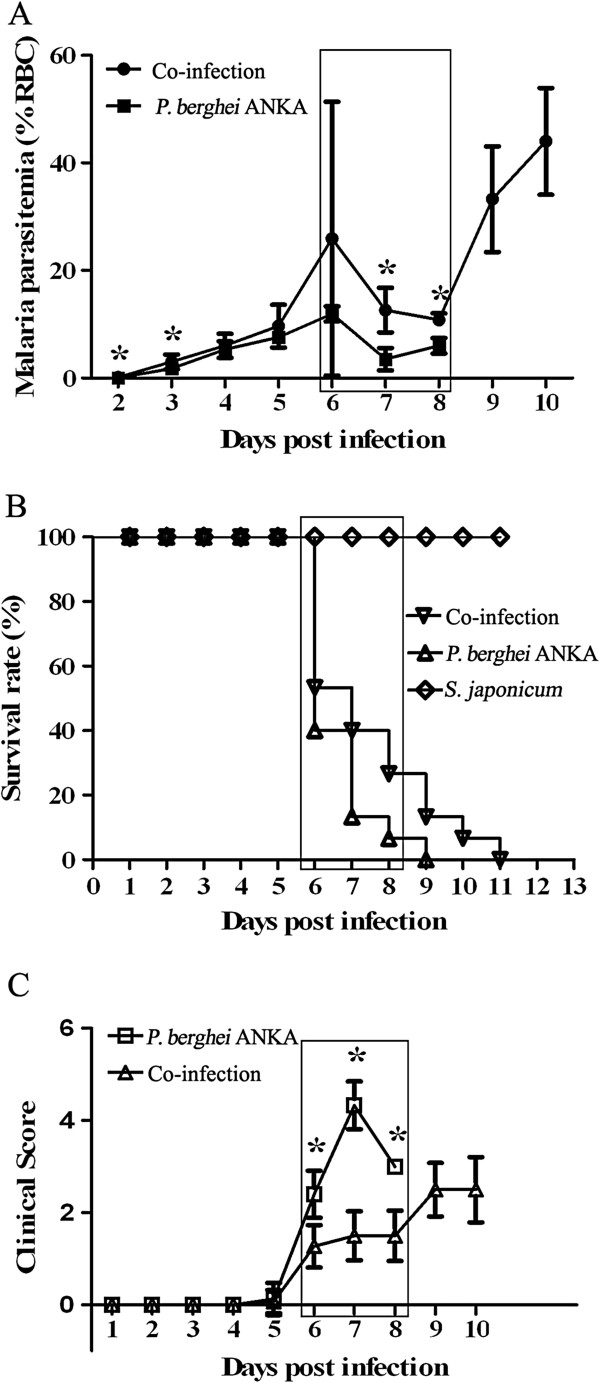
**Parasitaemia (A), survival rate (B), and clinical disease assessment by clinical score (C) in C57BL/6 mice infected with *Schistosoma japonicum *and then inoculated with 1**×**10**^**6 **^***Plasmodium berghei *ANKA-pRBCs eight weeks later (co-infection) and were analysed together with *P. berghei *ANKA-mono-infected mice (*P. berghei *ANKA).** For monitoring the malaria parasitaemia, a Giemsa-stained thin smear was made daily. The open box indicates the susceptible period for CM. Values represent the means with SD (n=3 mice per group). Student *t* test by comparisons between the two groups was performed with * indicating *P*<0.01.

Malaria parasitaemia (Figure 1A), mortality (Figure 1B) and ECM scores (Figure 1C) were recorded daily in the *P. berghei* ANKA-mono-infection mice and the co-infection group mice and comparisons of these were made between the 2 groups. During the challenge, the mean parasitaemia of the mice in both groups increased except for a transient decline that was observed on day 7 post-infection (pi). The co-infected mice had a significantly higher level of parasitaemia than *P. berghei* ANKA-mono-infected mice on day 2, 3, 7 and 8 pi. Between day 6 and day 8 (the period of susceptibility to CM), most of the mice presented clinical signs of ECM and subsequently died. Mice in the *P. berghei* ANKA group had significantly higher scores, based on the clinical scores used for assessment of clinical ECM symptoms, than mice in the co-infection group on day 6, 7, and 8. This indicated that *P. berghei* ANKA-mono-infected mice had more severe cerebral pathology than did the co-infected mice. The survival rate of mice in the co-infection group was slightly higher than that in the *P. berghei* ANKA mono-infection group during the period of susceptibility to CM, but this was not significant. All animals died on day 9 and day 11 for *P. berghei* ANKA-mono-infected and the co-infected group, respectively.

All mice in the *S. japonicum-*mono-infected group survived until day 11.

### Cytokine concentrations

As this study aims to evaluate the effect of a pre-existing *S. japonicum* infection on a following malaria infection, changes in corresponding cytokines were monitored on days 0, 3, 5 and 8, post-*P. berghei* ANKA infection, in the co-infected, *P. berghei* ANKA-mono-infected, and in the *S. japonicum-*mono-infected mice. Because the mice in the *S. japonicum-*mono-infected group were not received any other treatments during this period, no changes were observed in the levels of the cytokines that were measured. Data from *S. japonicum-*mono-infected mice are presented in the following diagrams, however data was only compared between co-infected and *P. berghei* ANKA-mono-infected mice.

**Figure 2:**
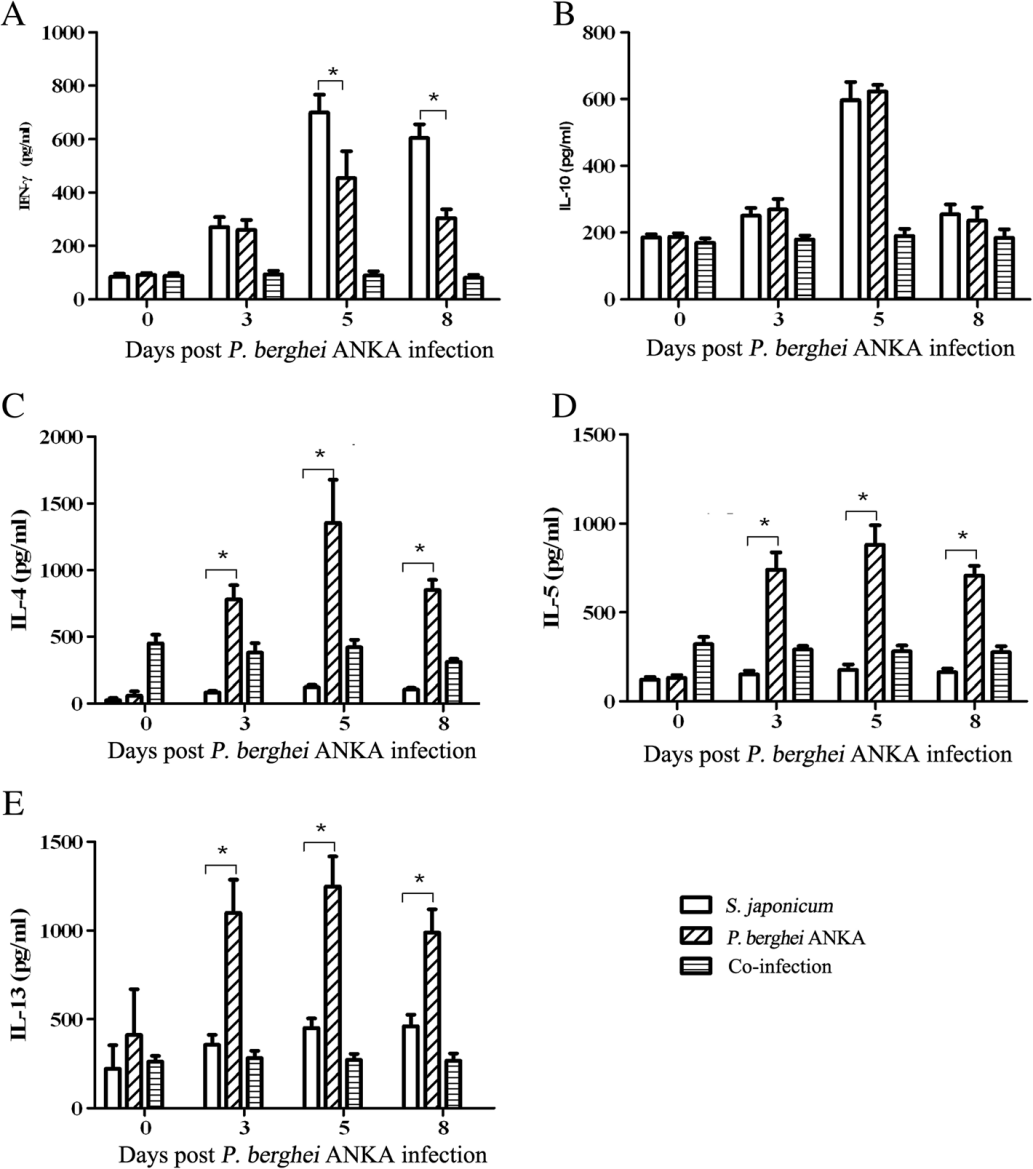
**C57BL/6 mice infected with *Schistosoma japonicum *and then inoculated with 1×10**^**6 **^***Plasmodium berghei *ANKA-pRBCs eight weeks later (co-infection) and were analysed together with *P. berghei *ANKA-mono-infected mice (*P. berghei *ANKA).** The mice were dissected on days 0, 3, 5, 8 post*-P. berghei* ANKA infection and splenocytes were cultured. Cytokines in the splenocyte culture supernatants were measured in duplicated wells using ELISA kits (R&D Systems, Minneapolis, MN, USA) according to the manufacturer's instructions. The bar charts represent the level of splenic IFN-γ **(A)**, IL-10 **(B)**, IL-4 **(C)**, IL-5 **(D)**, and IL-13 **(E)** from at least three mice per group. Bars represent the mean values ± SD. Student *t* test by comparisons between the two groups was performed with * indicating *P*<0.01.

One pro-inflammatory cytokine (IFN-γ) and four anti-inflammatory cytokines (IL-4, IL-5, IL-10, and IL-13) that were present in the supernatants of cultured splenocytes were measured by ELISA assay. This was done to evaluate the relationship between the levels of pro- and anti-inflammatory cytokines in the co-infected and *P. berghei* ANKA-mono-infected mice and to compare the levels between the two groups. Both the pro-inflammatory cytokine and the anti-inflammatory cytokines began to increase post- *P. berghei* ANKA infection, peaking on day 5 pi and then declining on day 8 pi. Compared with *P. berghei* ANKA-mono-infection group, the co-infection group showed a significantly lower level of IFN-γ on day 5 and day 8 pi and significantly higher levels of IL-4, IL-5 and IL-13 on day 3, 5, 8 pi. No difference in the level of IL-10 was found when comparing the two groups (Figure 2).

**Figure 3:**
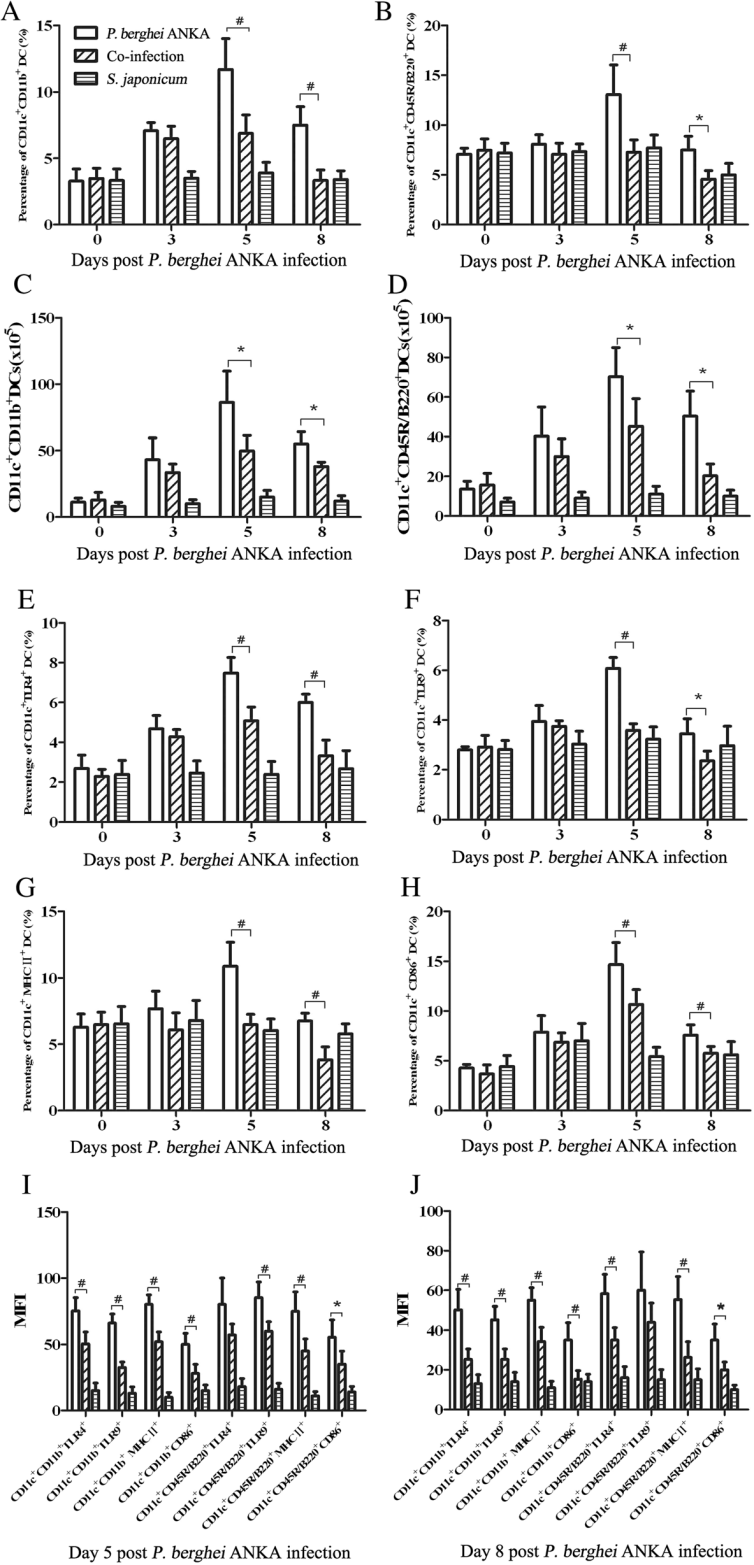
**Mice in *Plasmodium berghei *ANKA-mono-infection group and *Schistosoma japonicum-P. berghei *ANKA-co-infection group were compared at different time points according to data obtained by flow cytometry.** Proportion of splenic DCs subpopulation, CD11c^+^CD11b^+^ and CD11c^+^CD45R⁄ B220^+^, were analysed in **A** and **B**, with absolute number of these DCs present in **C** and **D**. Proportion of splenic CD11c^+^ DCs expressing TLR4 , TLR9, MHC class II, and CD86 of the two groups were compared in **E**, **F**, **G**, **H**, respectively. MFI of TLR4, TLR9, MHC class II, and CD86, which were expressed on CD11c^+^CD11b^+^ DCs and CD11c^+^CD45R⁄ B220^+^ DCs were present in **I** and **J**. At each time point, at least three mice per group were sacrificed. Bars represent the mean values ± SD. Student *t* test by comparisons between the two groups was performed with * indicating *P*<0.05 and # indicating *P*<0.01.

DC subpopulations were defined as CD11c^+^CD11b^+^ and CD11c^+^CD45R⁄ B220^+^ cells by flow cytometry and the change in subpopulations of splenic DCs in the co-infected mice *vs P. berghei* ANKA mono-infected mice were compared. Both the proportion/absolute cell numbers of CD11c^+^CD11b^+^ and CD11c^+^CD45R⁄ B220^+^ began to increase post-*P. berghei* ANKA infection, peaking on day 5 pi*.* and then declining on day 8 pi*.* The co-infected mice had significantly lower percentages/cell numbers of both CD11c^+^CD11b^+^and CD11c^+^CD45R⁄ B220^+^ on day 5 and day 8 pi, respectively when compared with *P. berghei* ANKA-mono-infected mice (Figure 3).

The percentage of splenic DCs expressing TLR4, TLR9, MHC class II, and CD86 in the co-infected *vs P. berghei* ANKA-mono-infected mice was also compared. Both the proportion of CD11c^+^TLR4^+^, CD11c^+^TLR9^+^, CD11c^+^MHCII^+^ and CD11c^+^CD86^+^ began to increase post-*P. berghei* ANKA infection, peaking on day 5 pi and then declining on day 8 pi. The percentage of CD11c^+^TLR4^+^,CD11c^+^TLR9^+^, CD11c^+^MHCII^+^and CD11c^+^CD86^+^ were both significantly lower in the co-infected mice on day 5 and day 8 pi when compared with *P. berghei* ANKA-mono-infected mice (Figure 3). In addition, MFI of TLR4, TLR9, MHC class II, and CD86, which were expressed on CD11c^+^CD11b^+^DCs and CD11c^+^ CD45R⁄ B220^+^ DCs were shown on Figure 3.

**Figure 4:**
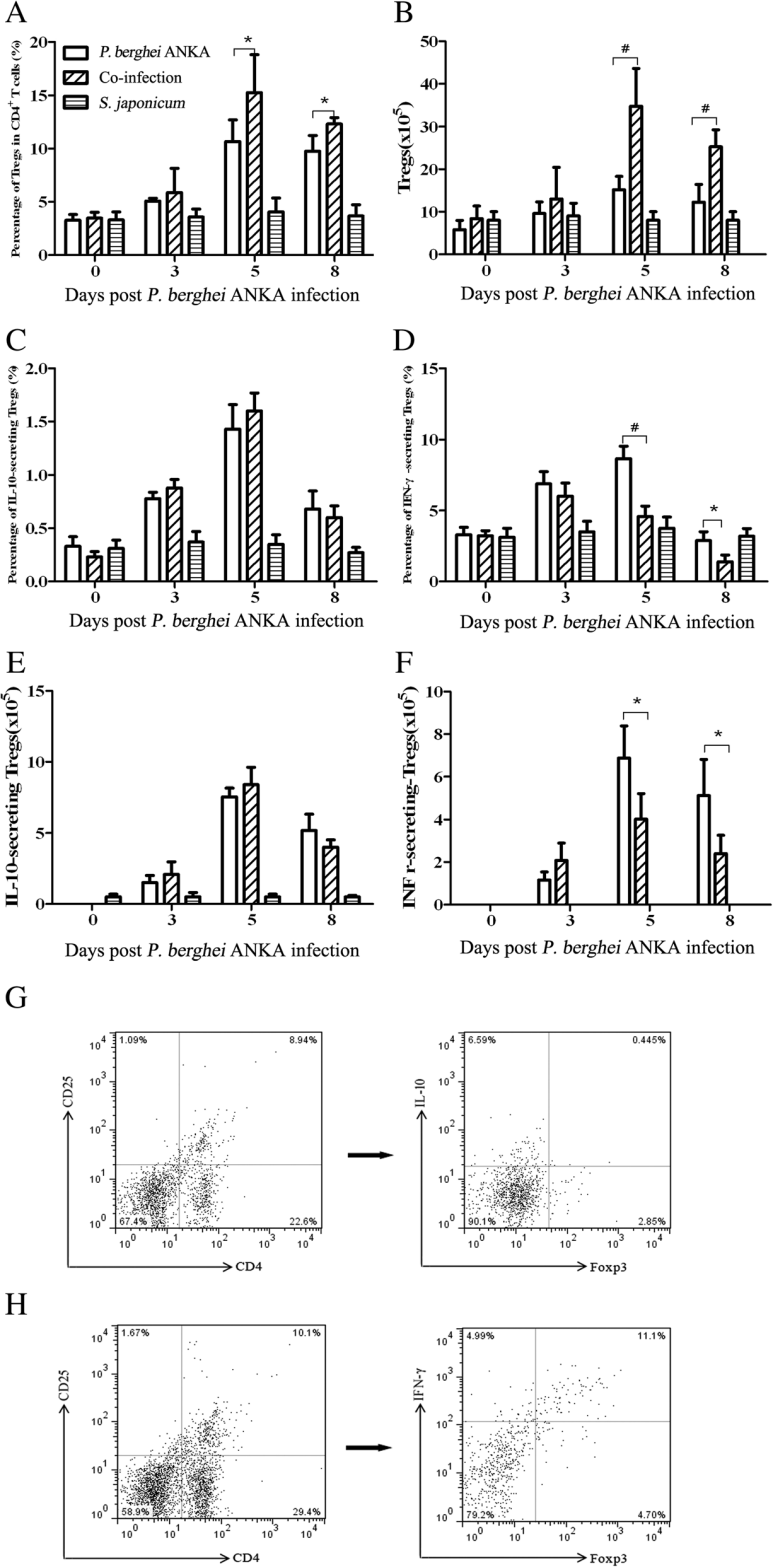
**Mice in *Plasmodium berghei *ANKA-mono-infection group and *Schistosoma japonicum-P. berghei *ANKA-co-infection group were compared at different time points according to data obtained by flow cytometry.** Proportion and absolute number of CD4^+^CD25^+^Foxp3^+^Tregs, IL-10-secreting Tregs, and IFN-γ-secreting Tregs were analysed in **A**, **B**, **C**, **D**, **E**, **F**, respectively. Gating strategy for identifying splenic Tregs were shown on **G** and **H** by representative dot plots. At each time point, at least three mice per group were sacrificed. Bars represent the mean values ± SD. Student *t* test by comparisons between the two groups was performed with * indicating *P*<0.05 and # indicating *P*<0.01.

The kinetics of Tregs in both groups of mice was followed by flow cytometry to evaluate the role of Tregs in the response to malaria infection and to compare of the proportion/absolute number of Tregs between co-infected and *P. berghei* ANKA-mono-infected mice. Tregs in the spleen CD4^+^ T-cell population increased after *P. berghei* ANKA infection with peaks appearing on day 5 pi and then declining. Also, the proportion/absolute number of Tregs on day 5 pi and day 8 pi was significantly higher in the co-infected mice than in the *P. berghei* ANKA-mono-infected mice (Figure 4).

Cytokine-secreting-Tregs in co-infected and *P. berghei* ANKA-mono-infected mice were also compared. The proportion/absolute number of IL-10-secreting-Tregs was slightly higher on day 5 pi, but then slightly lower on day 8 pi in the co-infected mice than in the *P. berghei* ANKA-mono-infected mice, however these differences were not significant. The proportion/absolute number of IFN-γ-secreting Tregs was significantly lower in co-infected mice than in *P. berghei* ANKA-mono-infected mice on day 5 pi and day 8 pi (Figure 4).

**Figure 5:**
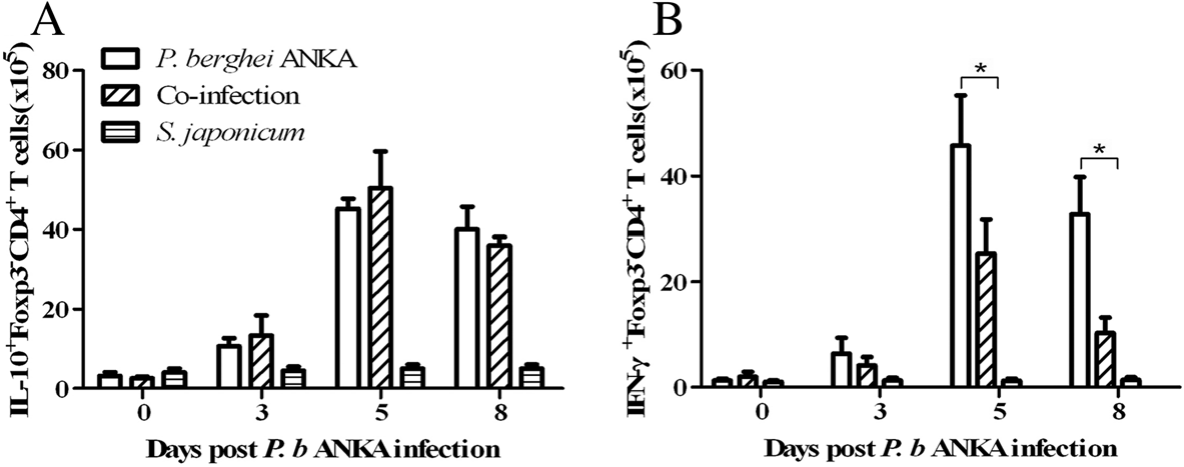
**Absolute number of IL-10**^**+**^**Foxp3**^**-**^**CD4**^**+ **^**T cells (A) and IFN-γ**^**+**^**Foxp3**^**-**^**CD4**^**+ **^**T cells (B) were compared between the *Plasmodium berghei *ANKA-mono-infection group and *Schistosoma japonicum-P. berghei *ANKA-co-infection group at different time points.** At each time point, at least three mice per group were sacrificed. Bars represent the mean values ± SD. Student *t* test by comparisons between the two groups was performed with * indicating *P*<0.01.

Finally, the IFN-γ and IL-10 produced by the Foxp3^-^CD4^+^ T cells in co-infected and *P. berghei* ANKA-mono-infected mice were compared. There was a significantly higher level of IFN-γ^+^Foxp3^-^CD4^+^ in the co-infected mice when compared with *P. berghei* ANKA-mono-infected mice, while the level of IL-10^+^Foxp3^-^CD4^+^ remained unchanged in both groups (Figure 5).

### Histopathology

**Figure 6:**
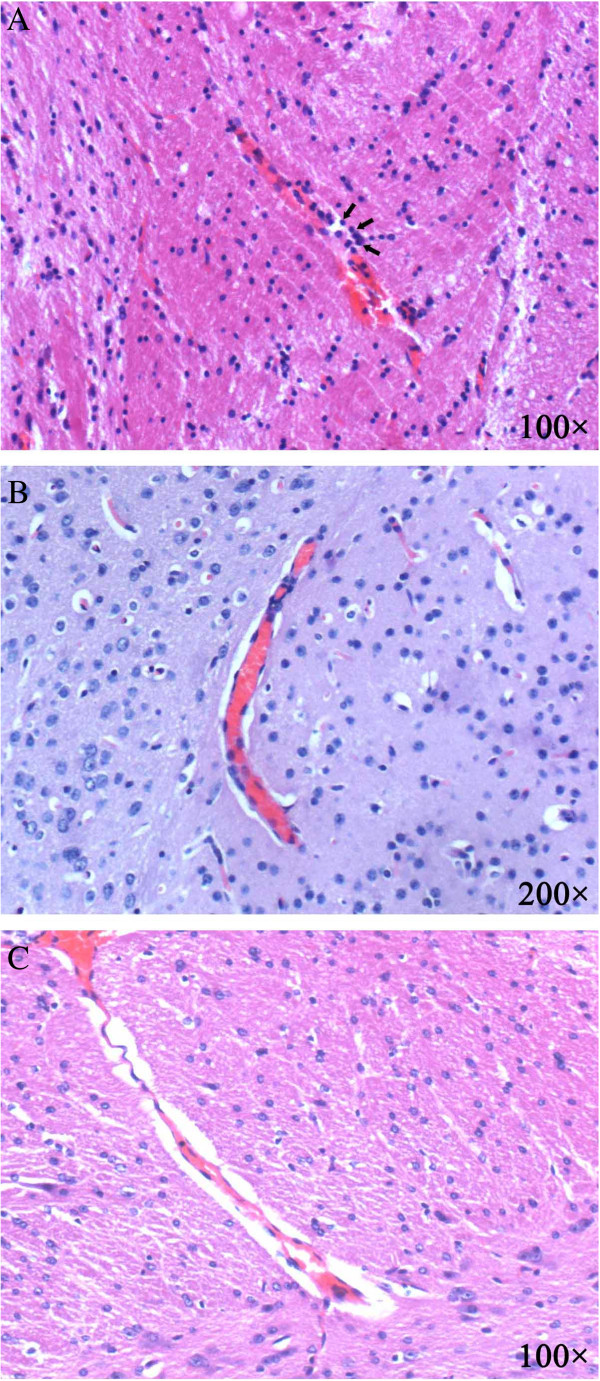
**Brain sections were obtained from *Plasmodium berghei *ANKA-mono-infected and *Schistosoma japonicum-P. berghei *ANKA-co-infected mice on day 8 post-*P. berghei *ANKA infection.** Conspicuous haemorrhage and mononuclear cell accumulation was observed in the *P.b* ANKA-mono-infected mice **(A)**. In contrast, the co-infected mice exhibited intact brain vessels **(B)** or only mild vessel wall oedema **(C)**. Sections stained with H&E.

Histopathology sections from co-infected and *P. berghei* ANKA-mono-infected mice, that had been euthanized on day 6, 7, and 8 post-infection, were analysed to evaluate the effect of pre-existing *S. japonicum* infection on *P. berghei* ANKA induced brain histology. Two normal animals were also euthanized to serve as a control. Conspicuous haemorrhage and mononuclear cell accumulation was observed in all of the *P. berghei* ANKA-mono-infected mice (Figure 6A). In contrast, most of the co-infected mice exhibited no signs of brain pathology (similar to the normal controls). The brain vessel wall was intact and no immune cells were seen beside the vessel (Figure 6B). Mild vessel wall oedema was observed in two co-infected mice (Figure 6C).

## Discussion

Many studies have been carried out to examine the impact of helminths on malaria infection, both in mouse models and in humans, yet the results are limited and often conflicting. Immune responses to parasitic infections are complex with many cytokines potentially playing crucial roles in the Th1-like or Th2-like responses. It is well known that helminth infections can ameliorate pro-inflammatory conditions, partly due to an inherent ability to induce Th2 responses [8-10]. In the current study, pre-existing *S. japonicum* infection enhanced Th2 responses to malaria infection, as evidenced by higher levels of IL-4, IL-5 and IL-13 that were present in the co-infected mice. Also, the co-infected mice had lower levels of IFN-γ, which indicates a suppressed Th1 response to malaria infection. These cytokine changes may suggest a polarized Th2 response to malaria infection when concomitant with *S. japonicum*. The histopathology findings supported this point as well since no changes, or only mild brain pathology, developed in the co-infected mice.

It is generally considered that Tregs are involved in the regulation of the immune response to malaria infections while the potential to determine disease outcome remains unclear. A previous report demonstrated that Tregs were required to limit pro-inflammatory immune responses in BALB/c mice in order to prevent ECM during secondary infections [24]. It has also been reported that Tregs can inhibit the Th1 immune response via modifying DCs and inducing the production of IL-10 during *P. yoelii* 17XL infection [25,26]. It has also been confirmed that the occurrence of Tregs during *P. berghei* ANKA infection is negatively associated with the production of IFN-γ [22]. Induced and/or activated Tregs may be beneficial to the host in malaria (when parasitaemia is well-controlled) by down-regulating the inflammatory response and thereby preventing immune-mediated pathology. On the other hand, Tregs could hamper the responses required for initial control of parasitaemia, permitting unbridled parasite growth if Tregs exerts its suppressive effects too soon, leading to severe disease [27]. In the current study, up-regulation of Tregs in the co-infected mice, indicated a pre-existing *S. japonicum* infection had strengthened the Th2 responses to malaria infection; this may protect against immunopathological impairment caused by Th1 responses. It is possible that the increased Tregs in the co-infected mice caused an enhanced Th2 response, which impeded the clearance of protozoan since significantly higher malaria parasitaemia (on day 7 and 8 post-*P. berghei* ANKA infection) existed in the co-infected mice and there was no significant difference in survival between *S. japonicum*-*P. berghei* ANKA-co-infected and *P. berghei* ANKA-mono-infected mice on day 6 - day 8 pi. Thus, it appears that there was a balance between both positive and negative effects, on the malaria infection, due to the increased Tregs.

Immune activity of Tregs can be mediated by a cell-contact-dependent mechanism and through the secretion of suppressive cytokines such as IL-10 [28,29] and promoting cytokines such as IFN-γ. It has been previously demonstrated that the proportion of IL-10-secreting Tregs is consistent with the Tregs population in *P. y* infected mice and that the immune-suppressive activity of Tregs can be mediated through the secretion of IL-10 [25]. In the current study, the lower level of IFN-γ-secreting Tregs found in the co-infected mice indicated a weakened Th1 response to malaria infection. As the majority of the IL-10/ IFN-γ-producing T cells appear to be Foxp3^-^, and thus constitute inducible Treg cells, the level of IL-10^+^/IFN-γ^+^-Foxp3^-^CD4^+^ was further analysed and these results also indicated a suppressed Th1 response in the co-infected mice.

Recent studies have indicated that helminths also have direct anti-inflammatory effects on innate immune responses. Kane *et al.*[10] confirmed that the eggs of *S. mansoni* could suppress LPS-induced activation of immature murine DCs, including MHC class II. As specialized antigen-presenting cells (APCs), DCs play an important role in the activation of T cells and consequently in the induction of adaptive immune responses [30]. These cells are classified into two main subpopulations [31]: myeloid DCs (CD11c^+^CD11b^+^) and plasmacytoid DCs (CD11c^+^CD45R/B220^+^). Various patterns of proliferation may be manifested by these DCs when stimulated by different pathogens. TLRs are expressed on or within innate immune cells, including DCs, and recognize pathogen-associated molecular patterns from different microorganisms [32]. Accumulating evidence suggests that TLRs are also involved in immune responses to protozoan parasites [33-35]. It has been reported that TLR9 responds to haemozoin [33,34] and that TLR4 responds to the putative toxin glycosylphosphatidylinisitol (GPI) from *Plasmodium falciparum*[36], resulting in the production of cytokines and chemokines, as well as up-regulation of co-stimulatory molecules [33]. Polymorphisms in TLR9 and TLR4 are associated with disease manifestation [37,38]. Stimulation of T-cell responses and more importantly, induction of Th1/Th2 cell development, is associated with maturation of DCs as well as production of Th1/Th2 cytokines [39-41]. Up-regulation of MHC class II and co-stimulatory molecules (i e CD80, CD86) are characteristic of the maturation of DCs [39-41]. High expression of MHC class II molecules is crucial for DCs to present antigens to CD4^+^ Th cells. A previous report has confirmed that the bulk of Th1/Th2 responses are present when the CD80/CD86 signaling pathway is blocked [42].

Ing *et al.*[43] demonstrated that DCs selectively phagocytose *Plasmodium*- pRBCs and present pRBC-derived antigens to CD4^+^ T cells *in vitro*, suggesting that DCs may play a primary role as APCs in malaria infections. It has been shown that DCs are involved in the establishment and regulation of T-cell-mediated immunity in mice infected with malaria [20] and that blood-stage *P. yoelii* 17XL infection induced increased numbers of splenic CD11c^+^DCs positive for MHC class II, CD80 and CD86, which is consistent with the establishment of the Th1 immune response [20]. In the current study, the effect of pre-existing *S. japonicum* infection on malaria infection was further evaluated by analysing distinct DC responses. Results indicated that pre-existing *S. japonicum* infection had an anti-inflammatory effect during the following malaria infection by directly suppressing the ability of DCs to produce pro-inflammatory mediators.

Bucher *et al.*[5] and Waknine-Grinberg *et al.*[4] found that a concomitant *S. mansoni* infection conferred protection against brain pathology caused by ECM, which is consistent with results from the current study. Tregs and the associated cytokines were further examined in the current study to investigate the immunomodulatory mechanism involved in the process of co-infections since these cytokines are important for establishing Th2 responses. In addition, assessment of DC responses during co-infection facilitated a better understanding of the direct anti-inflammatory effects a pre-existing schistosome infection has on the innate immune responses to malaria infection.

### Study limitations

This was an acute study, conducted over an eight-day period, in mice that had been exposed to *plasmodium* infection after being infected eight weeks prior with *S. japonicum*. This means that the immune responses to *S. japonicum* would the strongest, before modulating to the chronic phase around week 12 post-infection.

## Conclusions

The immune responses to malaria infection in *P. berghei* ANKA-*S. japonicum* co-infected mice were assessed and the changes in the cytokines, caused by pre-existing *S. japonicum* infection, were analysed. A Tregs-associated Th2 response played an important role in protecting ECM pathology. Pre-existing *S. japonicum* infection suppressed TLR ligand-induced DC maturation and had anti-inflammatory effects during malaria infection, not only by virtue of its ability to induce Th2 responses, but also by directly suppressing the ability of DCs to produce pro-inflammatory mediators.

## Abbreviations

CM: Cerebral malaria; DCs: Dendritic cells; ECM: Experimental cerebral malaria; GPI: Glycosylphosphatidylinisitol; IFNγ: Interferon-γ; IL-10: Interleukin-10; mAb: Mono-clone antibody; pRBCs: Parasitized red blood cells; P. berghei ANKA: *Plasmodium berghei* ANKA; RT: Room temperature; TLRs: Toll-like receptors; Tregs: Regulatory T cells.

## Competing interests

The authors declare that they have no competing interests.

## Authors’ contributions

All of the authors collaborated on the work presented in this study. M-lW, Y-mC and E-jL defined the research theme; M-lW prepared the infected animal models, designed the methods, performed the experiments, and interpreted the results; YZ and Y-jG analysed the data; and M-lW, YZ, E-jL and Y-mC drafted the manuscript. All authors read and approved the final version of the manuscript.
